# More Is More

**DOI:** 10.3201/eid1906.AC1906

**Published:** 2013-06

**Authors:** Polyxeni Potter

**Affiliations:** Centers for Disease Control and Prevention, Atlanta, Georgia, USA

**Keywords:** art science connection, emerging infectious diseases, art and medicine, James Barsness, My Valley, maximalism, More is More, the SARS experience, about the cover

**Figure:**
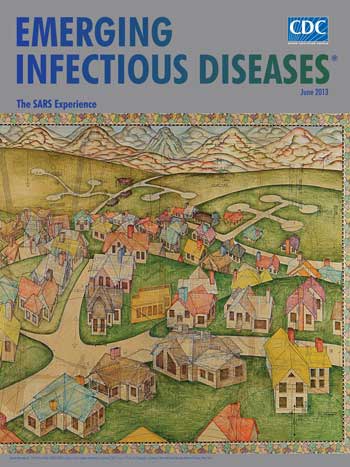
**James Barsness (b. 1954) *My Valley* (2003–2005) Acrylic, ink on paper mounted on canvas (125.7 cm x 171.5 cm)** Copyright courtesy of the artist and George Adams Gallery, New York

“For certain more curious and disenchanted spirits, the pleasure of ugliness comes from an even more mysterious sentiment, which is a thirst for the unknown and a taste for the horrible,” wrote Charles Baudelaire. “It is this sentiment, the germ of which all of us carry inside to a greater or lesser degree, that drives certain poets into clinics and anatomy theaters, and women to public executions.”

This taste for “ugliness” seems to also drive Jim Barsness’ exploits into the absurd and horrific, the landscape of nightmares rife with physical and moral decay. Taken to excess on the artistic canvas, ugliness and the absurd have been characterized as maximalism, a modern movement in literature and the arts that celebrates richness, decoration, sensuality, luxury, and fantasy. A genre that thrives on redundancy and overt accumulation, maximalism emphasizes a creative process or art-making that is also laborious and cumulative. “I use paper mounted on canvas because I like that sense of it being really hard to get the image ground down through all the layers of stuff. It becomes indelible.”

“The first time I did anything in art that had any profound consequences was to Draw Winky.” That was in sixth grade at a cartoon drawing contest. Sponsored by the Famous Artists Institute in Minneapolis, the contest was advertised on matchbook covers and comic books and invited children to draw a lumberjack or a baby deer. The drawing won Barsness a scholarship at the Institute’s correspondence school. His roots in colloquial drawing, doodling, and pop culture remain strong in many of his pieces, where comic strips provide the background for figures, showing his penchant for poking fun at sacred cows in society and in art. “My art partly has to do with my crackpot ideas about life and living.” These ideas lay out aspects of human behavior in mocking and irreverent scenes often filled with beastly or hybrid characters. “I try to hold myself to certain big ideas, but if something wants to go in another direction, I tend to let it go that way.”

“My most recent work is about finding, or knowing, my place,” the place where “we all belong” or fit in, where we know the rules, Barsness says. This all-important “place” is in various locations: the West, Bozeman, Montana, where he was born; Idaho and California, where he grew up and was educated; Athens, Georgia, where he lives and teaches art; Cortona, Italy, where he visits and works. Search for this metaphorical place also featured in Barsness’ decision to become an artist instead of following his other inclination, writing or storytelling. “It was a tossup. I wanted to be both.” Now “I can’t describe my drawings like stories. I start and try, but I can’t finish.” “I’m too distractible for the kind of complete narrative storytelling of, say, a novelist.” “And that’s the whole idea because I want people to supply the stories.” It is about story evoking rather than story telling.

“I’m interested in how groups of people interact in a continuous environment like a town,” Barsness explained in Monster’s Progress, a collection of his works. “It’s primarily about suppression of our natural interests for the good of the whole. I think it’s that moment of suppression that initiates much of our creative energy.” In exploring social interactions, Barsness is guided by psychology and mythology to create narratives with many layers and ramifications. He draws from eastern and western artistic traditions and taps multiple sources, from comic strips and fairy tales to Mughal miniature painting, from graffiti and medieval illuminated manuscripts to Tibetan sacred painting, from Pieter Bruegel the Elder and Hieronymus Bosch folk iconography to pop art. The result is humanity in a labyrinth of situations, puzzling, revealing, shocking.

“Ask a toad what beauty is, true beauty…. He will tell you that it consists of his mate, with her two fine round eyes protruding from her small head, her broad flat throat, her yellow belly, and brown back,” writes Umberto Eco in his book On Ugliness. The beholder or the situation, he argues, is judge. Situational ugliness is quite common. Along the same lines, imagine being in a familiar room, with a nice lamp sitting on the table. Suddenly the lamp floats upwards in midair. Though the room and the lamp are still the same, the situation has become unusual, disturbing. Something is wrong. This notion of the uncanny (the strange, the unsettling or sinister) is how Barsness creates the context for his ideas in *My Valley*, on this month’s cover.

“*My Valley* was inspired by my experience of growing up in suburbia, specifically Boise, where the new subdivisions are at the foot of the mountains, built right at the edge of a massive area of what’s left of wild America. But as I remember, there was absolutely nothing going on during the day in the suburbs. It was like a ghost town. There seemed to be nothing and nobody around.” Barsness captures the nothing and nobody in empty streets, ending in cartoonish cul-de-sacs and cookie cutter houses on their green lawns in the piedmont. This idyllic community, set against a stylized mountain range, casts colorful homes in suburban congeniality. Yet something is amiss. The inhabitants of the foothills are nowhere to be seen. The bland pleasantness of the homes and the monotony of space with its peculiar ambivalence fill the viewer with uneasiness.

Inside the painting’s intricate border, there is an eerie quiet. Uniformly shaded shutterless windows obscure the home interiors, but it is clear that no one is there, and this certainty adds a haunted quality to the scene. No children’s toys litter lane or lawn, and there are no vehicles. No neighbor looks over the fence nor old person peeks from behind a curtain. No dog or cat or squirrel in sight. An abandoned development or simply a bedroom community, this town is vacant. A seeming lack of substance gives the image its dramatic force.

Conversion of wilderness areas to agricultural or other commercially viable lands (housing, dams, mining) as a result of global socioeconomic and environmental changes in recent years adds a new angle to Barsness’ psychological profile of suburbia. Despite the periodic calm and emptiness, much is actually happening in the periphery of those seemingly vacant enclaves in the foothills: expanded demand for natural resources, deforestation, ecosystem disruptions, demographic pressures, increased urbanization, intensified crop and animal production, population movements. Expanding markets and farms bring diverse species together, facilitating exchange of microbes. The uncanny, so effectively captured in Barsness’ painting, becomes even more disquieting seen within a global context. Like the artist’s more traditional maximalist creations, this community on the edge of the wilderness is part of modern life’s complexity and intrigue.

As people move out of metropolitan areas unwittingly encroaching on the wilderness, they are exposed to and come in contact with wildlife. Animals move into human neighborhoods or are brought in through wildlife trade. And this neighborliness back and forth is not without consequences. Humans are exposed to wildlife microbes, which sometimes make the leap from animal to human hosts. The role of bats and civet cats in SARS, wild waterfowl in avian influenza A(H5N1), and infected birds in West Nile virus infection points to community‒wildlife interaction as an effective conduit for zoonotic microbes to enter new niches.

In *My Valley* as in many other works, Barsness includes elements for which he offers no explanation. “I like obsessive detail…. I come from the direction that everything is significant. You have to pretend that nothing matters while proceeding as if everything matters.” Fine, draft-like lines and arrows throughout the painting outline some subliminal complexity. These linear parameters, dotted and stamped in fine print and discreetly crisscrossing the canvas, invite metaphorical interpretation. Health emergencies in the past 50 years, among them flu pandemics, anthrax attacks, and a SARS outbreak, have prompted planning and emergency response efforts within the global public health community, a seamless underlying safety network against future crises.

In art, the tension generated by expansive inclusion of elements, the absurd and even ugliness and the uncanny, promotes understanding that may otherwise be lost. Likewise in public health, where an endless supply of unseen creatures, as monstrous and horrific as any found in science fiction, the art of Hieronymus Bosch, or Jim Barsness’ imagination, await the opportunity to wreak havoc. Extensive, even obsessive, public health planning is required. “More is more” takes on a new meaning as the wild, the repugnant, and the horrific, meet the banal and the fortuitous in nature.

## References

[1] Barsness J. You belong here: paintings and prototypes. New York: George Adams Gallery; 2004.

[2] Barsness J. Monster’s progress. Stockbridge (MA): Hard Press Editions; 2005.

[3] Eco U, editor. Translated by A. McEwen. On ugliness. New York: Rizzoli Publications; 2008.

[4] Horby P, Pfeiffer D, Oshitani H. Prospects for emerging infections in east and southeast Asia 10 years after severe acute respiratory syndrome. Emerg Infect Dis. 2013;19:853–60. 10.3201/eid1906.121783 23738977 PMC3713834

[5] Iskander J, Strikas RA, Gensheimer KF, Cox NJ, Redd SC. Pandemic influenza planning, United States, 1978‒2008. Emerg Infect Dis. 2013;19:879–85. 10.3201/eid1906.121478 23731839 PMC3713824

[6] Barsness J. Icons of comic relief. Spike Magazine. 2011 Sep 5 [cited 2013 Apr 15]. http://www.spikemagazine.com/james-barsness.php

[7] Koplan JP, Butler-Jones D, Tsang T, Yu W. Public health lessons from severe acute respiratory syndrome. Emerg Infect Dis. 2013;19:861–3. 10.3201/eid1906.121426 23739634 PMC3713823

